# Comprehensive Analysis of Applied Machine Learning in Indoor Positioning Based on Wi-Fi: An Extended Systematic Review †

**DOI:** 10.3390/s22124622

**Published:** 2022-06-19

**Authors:** Vladimir Bellavista-Parent, Joaquín Torres-Sospedra, Antoni Pérez-Navarro

**Affiliations:** 1 Faculty of Computer Sciences, Multimedia and Telecommunication, Universitat Oberta de Catalunya, 08018 Barcelona, Spain; aperezn@uoc.edu; 2 Algoritmi Research Center (CALG), Universidade do Minho, 4800-058 Guimarães, Portugal

**Keywords:** indoor, positioning, Wi-Fi, bluetooth, Wi-Fi radio map, machine learning

## Abstract

Nowadays, there are a multitude of solutions for indoor positioning, as opposed to standards for outdoor positioning such as GPS. Among the different existing studies on indoor positioning, the use of Wi-Fi signals together with Machine Learning algorithms is one of the most important, as it takes advantage of the current deployment of Wi-Fi networks and the increase in the computing power of computers. Thanks to this, the number of articles published in recent years has been increasing. This fact makes a review necessary in order to understand the current state of this field and to classify different parameters that are very useful for future studies. What are the most widely used machine learning techniques? In what situations have they been tested? How accurate are they? Have datasets been properly used? What type of Wi-Fi signals have been used? These and other questions are answered in this analysis, in which 119 papers are analyzed in depth following PRISMA guidelines.

## 1. Introduction

The use of outdoor positioning solutions using Global Navigation Satellite Systems (GNSS) technology, such as GPS, GALILEO or GLONASS, is commonplace. Their success lies in the fact that only one receiver (e.g., a cell phone) is needed to obtain the position. However, in closed places (buildings, tunnels, etc.) all of these systems fail, and are unable to obtain a position because the signal cannot penetrate the walls.

To obtain positioning in indoor environments, a technology different from GNSS is needed. Nevertheless, there is not currently an equivalent universal solution. However, in recent years, there has been important progress in many of the technologies used for indoor positioning, including Inertial Positioning [1], Bluetooth [2], Ultrasound [3], Visible Light [4], Wi-Fi [5], etc. These technologies can be applied either individually or together, in what is known as sensor fusion [6,7]. In addition to these “classical” technologies for indoor positioning, promising approximations have recently appeared, such as 5G [8] and Wi-Fi mmWave [9]. Among these possibilities, Wi-Fi-based solutions are very popular, mainly because the infrastructure required for their deployment is already available everywhere, and if it is not, it can be implemented easily and cheaply. For this reason, there are a large number of items based on this technology and the number is growing all the time. In the last few years, there has been a significant increase in the application of machine learning models to enhance the accuracy of indoor positioning. This large volume of works requires a compilation, ordering, and classification of the results in order to assist researchers in selecting appropriate machine learning models for positioning purposes.

Thus, this work has two main contributions: (1) a review of papers published between 2016 and 2021 that use machine learning for indoor positioning, reporting information about the algorithms used, type of article (experimental/simulated), number of Access Point (AP) used, number of radio map reference points used, results obtained, type of signal used, and use or non-use of rooms in experiments; and (2) an analysis of how the main dataset, UJIindoorLoc, has been used in those papers along with the main drawbacks detected when using datasets. The selection of papers in this review was performed following the PRISMA guidelines [10].

This article is an extension of a work presented at the 2021 International Conference on Indoor Positioning and Indoor Navigation (IPIN 2021) [11]. Its novel contents include the following:

- The current work extends the analyzed period to the last five years, analysing a total of 119 published research works, 57 more than in [11];
- An analysis of solutions based on Artificial Neural Networks (ANN), Suport Vector Machines (SVM), and Random Forest (RF) is included;
- A comprehensive analysis of the most widely used public datasets (radio maps) and how they have been integrated in experiments performed by the research community;
- A discussion of the size of the operational areas considered in experiments performed in the reviewed works;
- Extended context, discussion, and conclusions.

The rest of this review is organized as follows. Section 2 reviews the existing literature related to indoor positioning. Section 3 describes the methodology used in this paper. Section 4 presents the detailed results in the form of a table. The results of the table are analyzed in Section 5. Finally, Section 6 provides conclusions.

## 2. Related Work

There are many articles based on Wi-Fi and machine learning algorithms. We found several reviews on this issue, although they answer different questions than those addressed in the current work. For example, [12] is a complete analysis of different indoor positioning articles, however, it is focused on collaborative positioning methods. Collaborative technologies rely on information exchange between different users and/or devices to improve overall performance. The main advantage of this method lies in its infrastructure, which there is less of than in other methods, as well as its low maintenance requirements. Positioning is based on the calculation of data from various sources, such as users and devices, and therefore the main drawback is the need for additional computational resources.

In [13], the authors analyze different articles on indoor positioning; however, they do it at an individual level and do not show any classification or comparative table, although it is a good compilation of articles that use radio, light, or inertial technologies for indoor positioning.

Channel State Information (CSI) for positioning is the focus of the paper in [14], a survey that provides many resources on CSI-based indoor localization methods and includes state-of-the-art algorithms and systems. The authors include a comparative table with fourteen articles using this technology, although only few parameters are analyzed.

Regarding Visible Light Communication for indoor positioning, [15] provides a brief and useful review of ten papers that use machine learning algorithms and visible light solutions in their experiments.

In [16], the authors provide a summary and in-depth analysis of all the wireless technologies used in the field of indoor positioning. Thus, the authors consider works based on the received signal (RSSI or CSI) as well as works that use data such as Time Of Flight (ToF), Angle of Arrival (AoA) or Phase of Arrival (PoA). Their paper includes a review of the different methods used to achieve positioning, such as fingerprinting, multilateration, and triangulation. Finally, they classify the most widely used Machine Learning algorithms and the methods used to filter the received signals. However, it is important to note that the paper is a review of the technologies used, and does not analyze the contributions to the state-of-the-art of every paper individually.

In a paper similar to the previous one, Obeidat et al. [17] review systems not based on radio signals. Thus, the paper reviews positioning through any type of wireless signal as well as optical or magnetic solutions. The authors analyze the different algorithms and techniques used to achieve positioning; however, as in the previous article, they do not sort every paper individually.

Closer to the current review is [18] a survey centered on machine learning algorithms. In this paper, the authors present a compilation of articles based on the application of machine learning algorithms applied to different indoor positioning solutions and classify them by the type of algorithm used. Finally, the authors make a comparative table in which readers can decide which type of algorithm to choose depending on their specific positioning needs (low computational cost, precision, etcetera).

An extensive survey of machine learning techniques for indoor localization and navigation systems is provided in [19], including a deep analysis of all existing algorithms used in this field. The paper is focused on both the algorithms themselves and on different techniques to improve results while working with those algorithms (Data Preprocessing, Interpolating Missing Data, Filtering, etc…). The paper includes reviews of public datasets, performance evaluation parameters used, and other surveys.

Finally, Alhomayani et al. [20] narrow the scope and review fingerprint solutions jointly with deep learning algorithms. Their classification is contains a compilation of the most widely used public Wi-Fi radio maps and a short analysis of every one. However, as in the reviews mentioned previously, their review focuses more on the analysis of the different elements involved in indoor positioning than on analyzing the individual items, which is the focus of the current work.

Thus, as can be seen, many previous works have analyzed how positioning can be achieved using Wi-Fi; nevertheless, there are no previous works, to the best of our knowledge, that have analyzed which machine learning techniques are used, how they are tested, and how much every technique is used. In addition, none of the previous works have analyzed how public datasets (or radio maps) have been integrated into third party research works.

## 3. Methodology

In this work, in order to analyze the use of machine learning in Wi-Fi solutions to obtain position, the methodology that has been followed is that of a systematic review based on the PRISMA [10] guidelines. The three main steps of this methodology are: (1) to raise the research questions to set the objective of the review; (2) to look for the papers in the chosen digital databases that can answer the research questions; and (3) to establish a set of inclusion and exclusion criteria to finally keep only those papers that fit the research objective. These three steps drive the final selection of the articles that are part of this work.

The research questions are:

**RQ1.**  Which machine learning algorithms provide the best results in Wi-Fi-based indoor positioning?
**RQ2.**  What kind of Wi-Fi signal parameters provide the best results?
**RQ3.**  What are the most commonly used metrics in indoor positioning studies?
**RQ4.**  Are there substantial differences between simulated and experimental studies?
**RQ5.**  Which public radio signal maps are the most commonly used in simulations?

**Figure 1:**
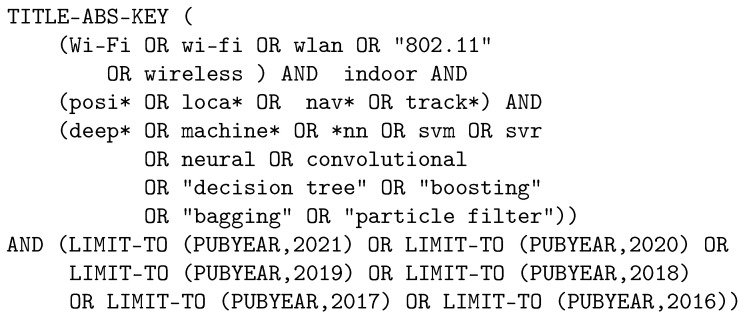
Query for *Scopus*.

**Figure 2:**
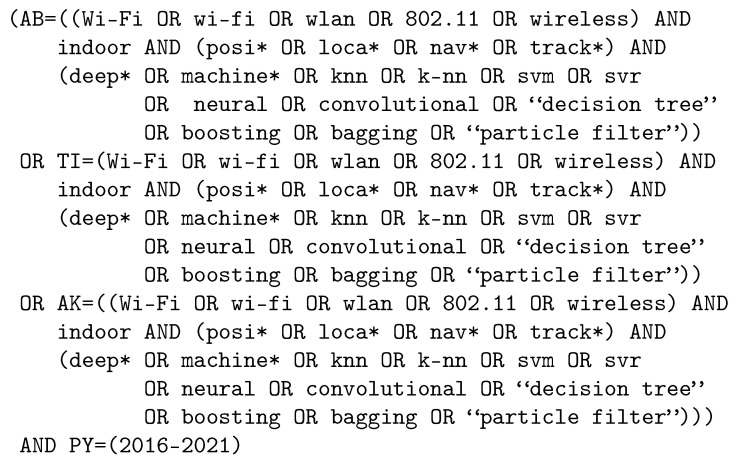
Query for *Web of Science*.

To perform queries, the *Web of Science* and *Scopus* databases have been chosen; these are reliable sources with sufficient content for an exhaustive review. Figure 1 and Figure 2 show the queries we have used to obtain the scientific papers in the two databases.

The inclusion criteria that selected papers must satisfy are:

**IC1.**  Written in English
**IC2.**  Coming from a conference or journal article
**IC3.**  Dealing with Wi-Fi-based positioning
**IC4.**  Positioning through Machine Learning algorithms
**IC5.**  Published between 2016 to 2021

The exclusion criteria are:

**EC1.**  Workshops and book chapters
**EC2.**  Positioning that is not 100% Wi-Fi or is based on Sensor Fusion
**EC3.**  Positioning that has part of the work outdoors
**EC4.**  Positioning based on classic multilateration (TOA, AOA, etc.)
**EC5.**  Positioning that uses a KNN-based algorithm or Particle Filter, as this is not considered Machine Learning

**Figure 3:**
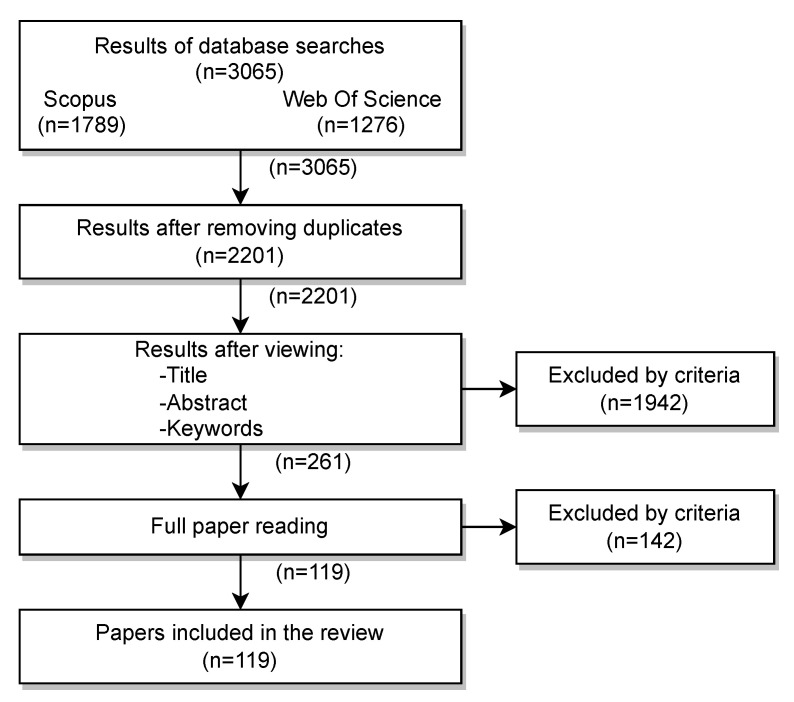
PRISMA flow diagram.

After the list of the papers had been obtained, the next step was to remove duplicates from all the results obtained from the two searches performed in *Web of Science* and *Scopus*. With the resulting articles, a first analysis of the title and abstract of each of them was carried out in order to rule out those which failed to meet the inclusion criteria or which met the exclusion criteria. Finally, a full reading was made of the included articles in order to verify whether they met the inclusion criteria. Those that were finally included were analyzed in answering the research questions. The diagram of the different results obtained in each step can be seen in Figure 3.

As can be seen, the original number of papers, after removing duplicates, was 2201. After reviewing them, 119 satisfied the inclusion criteria, and thus are the papers analyzed in the current work.

## 4. Results

This section presents the results obtained after the analysis of the 119 papers included in this review. The features analyzed regarding the research questions are summarized in Table A1, Table A2, Table A3 and Table A4, which are included in the Appendix. It is important to note the following items:

- Features not explained in the articles appear as N/A.
- Articles that include different experiments and/or simulations are grouped together.
- Articles that do not display a clear metric are marked in the column oError (Other Errors).
- Articles that are based on or use algorithms different from the main one are marked in the column sAlg (Secondary Algorithm)

The results shown in the tables are discussed and analyzed in the following section.

## 5. Discussion

In this section, we analyze the results from several points of view: the algorithms used, types of signals used, number of APs and reference points used, metrics, type of experimentation, and most commonly used radio maps.

### 5.1. Methods: Algorithms and Machine Learning Models

**Figure 4:**
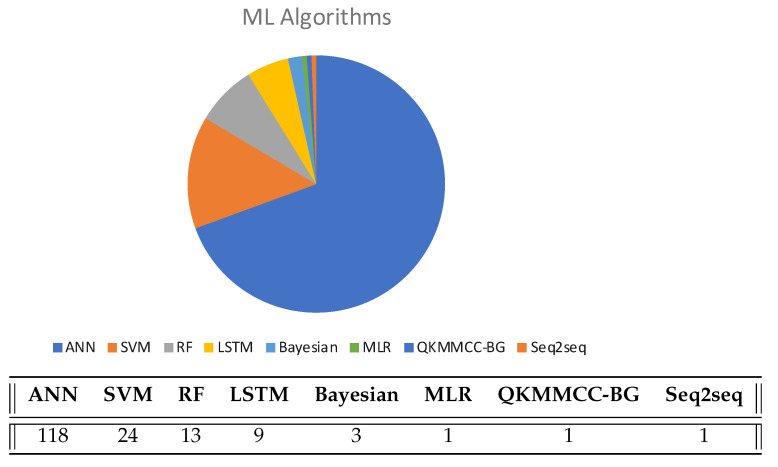
Most widely used algorithms and Machine Learning models.

Figure 4 shows the distribution of algorithms. From these results it can be seen that the most commonly used algorithms are those based on ANN. Specifically, there are up to 118 works (around 69% of the total analyzed works) that use this machine learning model or any of its variants (Deep reinforcement learning (DRL), Extreme learning machine (ELM), Convolutional Neural Networks (CNN), Deep Neural Networks (DNN), Back-Propagation Neural Network (BPNN), Capsule Neural Network (CapsNet), Stacked Denoising Autoencoders (SDA), Variational Autoencoder (VAE), Deep Belief Network (DBN), Recurrent Neural Networks (RNN), Multilayer Perceptron (MLP), Neural Network (NN), Single Multiplicative Neuron (SMN), and Deep Q-Networks (DQN)). Neuronal network-based algorithms are specially appropriate for nonlinear functions, and the fluctuating signal type of Wi-Fi fits perfectly into them.

The number of solutions based on ANN has been growing in recent years. In fact, by the year 2021, 20 out of 24 articles used ANN. In addition, the best result in the analyzed papers (mean error of 0.11 m) were obtained using a Deep Neural Network to process data from Wi-Fi mmWave signals [9]. However, as we will see later, these results alone do not indicate anything, as factors such as the size of the test area, number of APs, etc. affect these results.

Several papers focus on combining different algorithms in order to choose the one that provides the best results in a particular case [21,22,23], while other papers focus on processing data collected from APs [24,25,26,27]; finally, we found two papers [28,29] that relied on applying a double algorithm, one to approximate the location and another to detail it more precisely from the first approximation.

In the following subsections, we look more deeply into the specifications of NN, SVM, and RF.

#### 5.1.1. Neural Networks

Neural networks are made up of layers of interconnected nodes. Their scheme essentially consists of an input layer, one or more hidden layers, and an output layer. During the training phase, the output is compared with the predicted result and the obtained error is calculated. This error is then propagated through the hidden layers and the weights of the nodes are modified in order to obtain better results. This process is repeated to improve accuracy.

In the articles we analyzed, there is no standard optimum configuration. Researchers perform different tests until they obtain a result that satisfies the two desired properties of accuracy and computation time. In [30], the authors use six layers with 512 nodes in every layer to achieve an accuracy of 2.4 m. However, the authors of [31] instead use only four layers, without specifying the number of nodes, while in [32] only two layers of 50 nodes each are used.

Thus, there is no a standard configuration. Nevertheless, it is important to take into account the difficulty of finding an optimal configuration, as it is influenced by different variables, such as the type of scenario where the experiments are performed, its shape, whether or not there are obstacles, the number of APs used, etc.

#### 5.1.2. Support Vector Machines

The class of algorithms called Suport Vector Machines (SVM) is based on projecting the results on a plane divided into two parts and grouping the results in one of the two parts. Thus, we are talking about a classifier algorithm. In the papers we analysed, we found several different versions of the SVM algorithm. Ref. [33] shows an M-LS-SVM algorithm, which is characterized by the use of linear functions instead of the quadratic functions of the original SVM; the authors obtained an accuracy of 2.7 m. However, [34] used the SVM algorithm directly, obtaining an accuracy of 0.7 m in a similar scenario, and [35] used a SVM algorithm with CSI instead of Received Signal Strength Indicator (RSSI) and obtained an accuracy of 1.909 m in a simpler scenario with no rooms or obstacles.

#### 5.1.3. Random Forest

Random Forest (RF) algorithms are based on the construction of a large number of decision trees to create a learning model. Each decision tree decides a class and the most common class ends up being the final prediction of the model. Its use in indoor positioning has been decreasing, and in the year 2021 no articles were detected that used it. In 2020, there were only three articles that used it, and none of these used scenarios with obstacles to perform the experiments.

The best accuracy found with RF is 1.68, from Maung et al. [36] in 2020, in a space of $112\;m^{2}$. However, in 2018 the authors of [37] claimed an accuracy of 1.20 m in a space $75\;m^{2}$, and the authors of [38] obtained an accuracy of 0.4033 m in a space of $80\;m^{2}$. From these results it seems that RF is an algorithm suitable for small spaces.

#### 5.1.4. Comparison of Models

The use of one algorithm or another is determined by different factors, such as computational resources, the amount of data to process, and the type of infrastructure (rooms, tables, walls…) where a fingerprinting-based system is to be implemented.

If we focus on computational resources, RF requires fewer resources than SVM algorithms. In fact, SVM-based algorithms tend to be almost unusable on large datasets because the training complexity of SVM is highly dependent on the size of dataset used.

At the level of infrastructure complexity, the situation is similar. SVM algorithms work very well for mitigating the NLOS of signals; therefore, they are ideal in small and complex sites. On the other hand, NNs are more configurable, and their usage can be adjusted for better performance based on lower precision. If not much precision is needed and speed is preferable, the number of nodes and neurons involved in the network can be adjusted [39].

In the case of large spaces, RFs have an advantage over SVMs, because these algorithms are appropriate on models that have been clustered, which is helpful in large scenarios. It is important to note that RFs are Decision Trees optimized to work with large amounts of data. ANNs are particularly suitable in situations where there is noise and multipath propagation, as well as where there are a large number of APs [18].

In summary, in small spaces and with little computational capacity SVM is the best option, while in complex situations and with large datasets, RF and NN are more complex to implement; however, they are more adaptable due to their great configuration capacity.

### 5.2. Types of Wi-Fi Signal Parameters Used

The most commonly used indoor positioning parameter is based on the RSSI; 114 of the reviewed works used RSSI. The second most used Wi-Fi signal is CSI, which was used by 15 papers.

Wi-Fi is available in many indoor spaces nowadays and is an easily accessible parameter from any device, including mobile and wearable devices. In general, the results obtained with RSSI have an accuracy between 1 and 8 m.

Nevertheless, these apparent good results can be due to the design of the experiments; therefore, these accuracies cannot be generalized or expected in different environments. The elements that drive to these accuracies can be, among others: (1) experiments with small spaces without obstacles and with many reference points, thus avoiding the effect of signal loss when passing through walls and the multipath effect (as explained in [40]); or (2) experiments that use training and validation data with little difference in terms of time and space, or using the data used for training for validation, as in [41].

**Figure 5:**
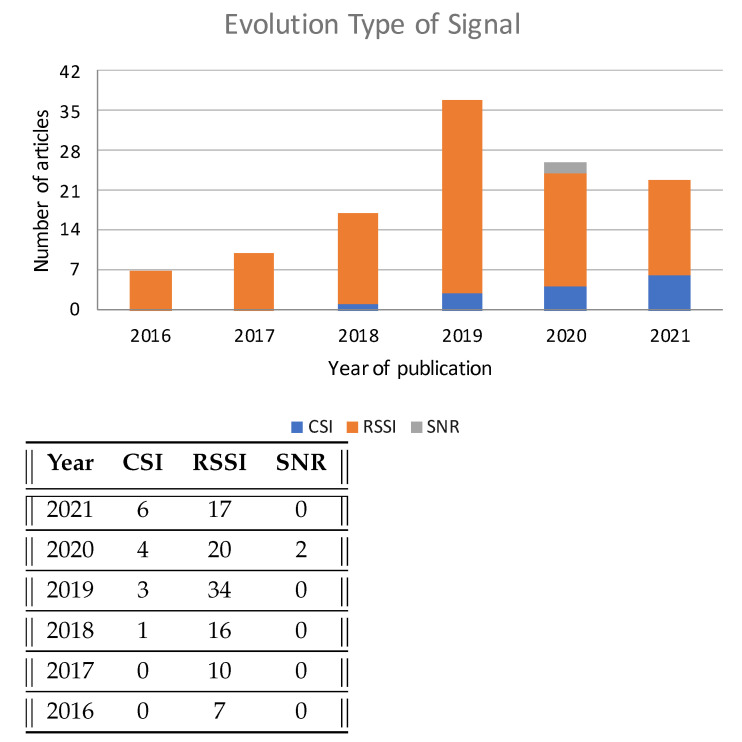
Evolution of the types of signal used.

On the other hand, there are 15 studies that use CSI [32,35,42,43,44,45,46,47,48,49,50,51,52,53] from a Wi-Fi signal, generally with better results in terms of accuracy than those obtained with RSSI. CSI is not widely used because the channel state information is not easy to obtain and requires specific network cards and modifications to the original *firmware* [52] (i.e., it cannot be used in smartphones). Despite this, we observed a large increase in the use of this parameter. Before the year 2020, only four papers used this parameter. However, in the last two years up to ten papers have used it, as can be seen in Figure 5. One reason for this may be that the RSSI parameter is reaching its limits, and new mechanisms are being explored as they are becoming more present in common everyday devices.

Finally, the Signal-to-Noise Ratio (SNR) parameter is beginning to be used, specifically in two papers [9,54], and in particular in combination with Wi-Fi networks that use *mmWave* instead of the classical networks that broadcast on traditional frequency channels, i.e., 2.4GHz and 5 GHz. SNR technologies show better positioning accuracy, and led to the best and the third-best results we found in this review.

### 5.3. Evaluation Metrics

In order to compare works among themselves, a common evaluation metric is needed. Most works report their results in terms of the average positioning error in different evaluation points, which is the positioning error defined as the Euclidean distance between the actual and estimated positions (*Mean Error* on the table). Among these, most report the Root Mean Squared Error (RMSE) as well. Other metrics used are the Mean Squared Error (MSE) and the Median Error. Another important metric is the percentage, which is used in one way or another in 36 papers [22,24,25,55,56,57,58,59,60,61,62,63,64,65]. Unfortunately, two articles do not show their results clearly; papers [66,67] only show a graph, however, it is difficult to determine the obtained results from the image.

**Figure 6:**
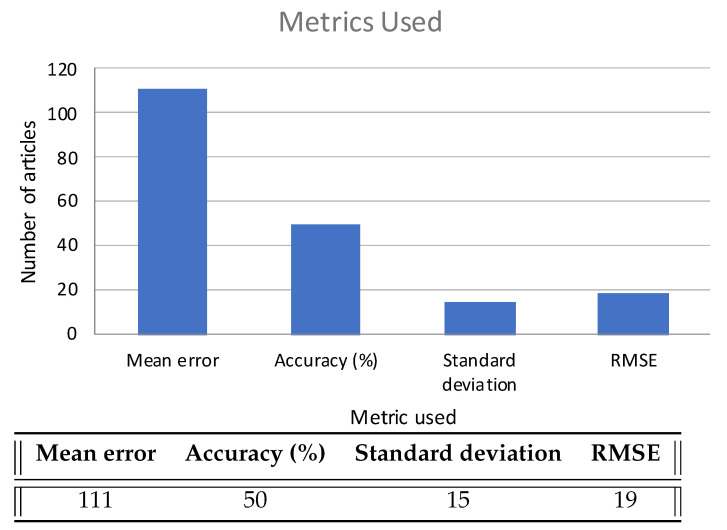
Metrics used.

In analyzing those papers that use metrics recommended by ISO/IEC 18305 [68] (the standard methodology to evaluate indoor localization systems), it can be seen that all of the articles (except those that do not show results) comply with this standard, specifically, mean error, accuracy in one zone or floor, root mean square error, and standard deviation. Figure 6 shows how many papers used each metric.

### 5.4. Experimental and Full Simulated Results

Regarding whether results were obtained experimentally or via simulation, we found seven papers that presented results from full simulations (with artificially generated data), 40 using public datasets, and 114 that presented empirical results (note that there were articles that perform several experiments and/or simulations).

Four of the papers reporting results based on simulations performed real-world experiments as well [29,45,69,70]. While the authors in [69,70] implemented a simple Log-Distance Path Loss (LDPL) model to generate the RSSI values, Ezzati Khatab et al. [45] included a wall attenuation factor to better model the radio propagation with the LDPL model under Non-Line-of-Sight (NLOS) conditions. In contrast to these, Bai et al. [29] used a more sophisticated Ray Tracing model to generate the RSSI values.

In any event, the results reported in simulations tend to be better than the ones reported in the real-world experiments performed in those papers that performed experiments in both scenarios. Those simulations assuming that Line-of-Sight (LOS) conditions are always met, included a low Gaussian noise, or implemented a simple model, represent an optimistic view of real-world evaluation in one way or another, and therefore the results may be much better in terms of positioning error. This is the case, for instance, in Zhang et al. [70], where the errors in the simulation are 30%–50% better than those reported in the real-world experiment.

**Figure 7:**
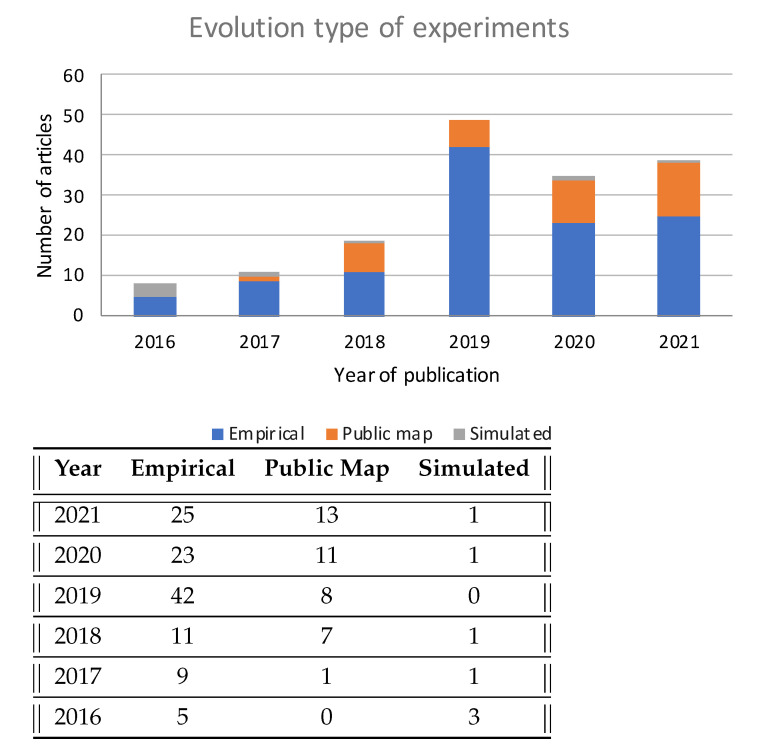
Evolution of experimental vs. simulated studies.

The empirical results reported are usually better than those obtained with public datasets. There are several reasons for this behavior. In general, researchers have much more knowledge about their own testing areas than those external areas included in public datasets, which impact the selection of the algorithm and its hyperparameters. Performing the experiments in their own facilities enables researchers to select an optimal sub-area for evaluation (e.g., the one with better Wi-Fi coverage or higher density of APs), have custom deployment of APs, or even add additional supporting infrastructure in the operational area. Therefore, public datasets are a more challenging testing scenario for algorithms; in addition, they allow for comparing different algorithms, as they are tested in the same area. Thus, it is important to note the increase in the number of papers that used public datasets in recent years, as can be seen in Figure 7.

### 5.5. Most Widely Used Public Datasets

Of the experiments reported from 2016, 22% were performed on public datasets; in 2021 this percentage rises to 39%, most likely due to the COVID-19 pandemic, although up to six papers from these years do not indicate what type of radio map they used. Thanks to these public datasets, researchers were able to provide useful results while continuing to perform experiments during the pandemic. In addition, these public datasets play a key role in research, as they allow researchers to compare different algorithms tested with the same data.

In the list, the most commonly used public signal map is UJIIndoorLoc [71] in all its variants (different buildings, any floor). It appears in 23 papers, and is clearly the most important, especially in the last two years, when the rest of the maps we found appear only once.

**Figure 8:**
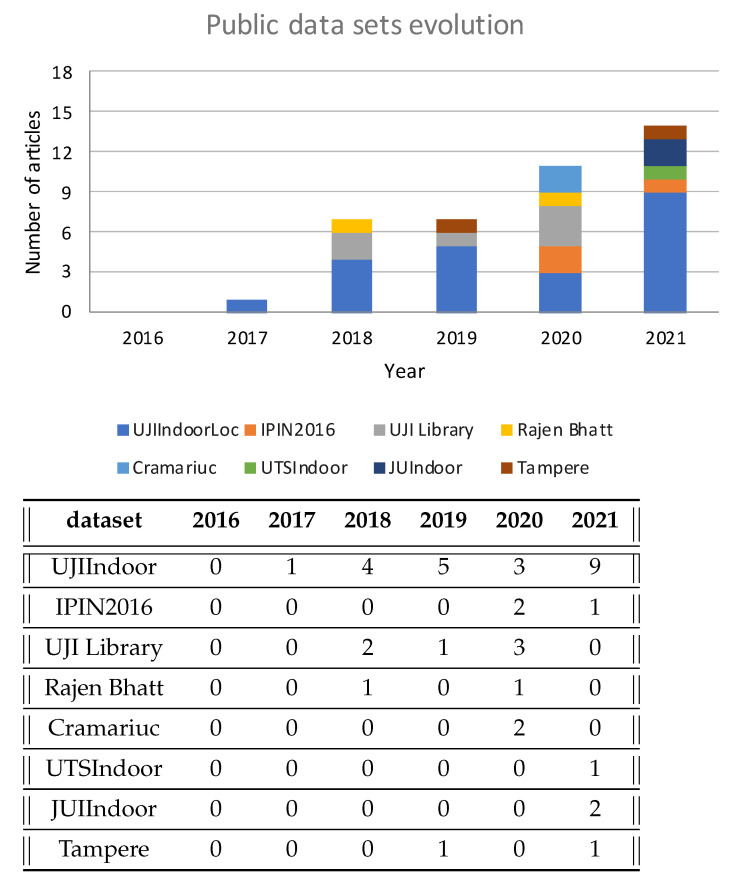
Evolution of the use of public datasets over the years.

Other radio-maps used are IPIN2016 [72], UTSIndoorLoc [73], JUIndoorLoc [74], Rajen Bhatt [75], Cramariuc [76], Alcala Tutorial 2017 (included in UJIIndoor), WIFINE [77], UJI Library [78], and Tampere [79]. In Table 1 we provide a summary of different attributes of the public datasets used, while Figure 8 shows the evolution of the use of different public datasets over the years. Note that before 2017 there were no public datasets that met the necessary conditions to be used in simulations, thus, we must recognize the recent contribution of these datasets to this field of research.

Because UJIindoorLoc is the most widely used dataset, we performed a deeper analysis into how it has been used by researchers.

#### UJIIndoor Results Analysis

Due to the large number of articles that performed their tests with UJIIndoorLoc, it is useful to provide a comparison of the different algorithms used on this dataset; results without the mean error have been omitted. Table 2 shows the main papers that used the UJIIndoorLoc dataset.

In analysing these papers, we detected different strategies in researchers’ methods of treating the original UJIIndoor data, resulting in a mean positioning error much below the baseline. From this analysis, we can conclude that the best result obtained by correctly using training and testing from UJIIndoorLoc is [84], with a mean error of 5.64 m.

Despite other works reporting lower positioning error results, these results cannot be directly compared to the baseline as their evaluation was restricted to a small area within the full operational area (a building and/or a floor) and/or the evaluation data contained samples from the original training set, as we show in the following paragraphs. In [41], the author separates the multi-floor and single-floor data to treat them independently, then, from the same dataset, separates 80% for training and 20% for testing. This method can lead to better results, as it is very likely that data taken at almost the same time can be in both the training and test sets. On the contrary, in [80], the authors use the validation component (1111 samples) of the dataset as the test set. The training portion of the dataset is split into training (15,950 samples) and validation (3987 samples) subsets based on an 80:20 split. This is a good practice, as the authors do not mix training and test data.

In [82], the authors selected a subset of the original dataset. In this case, the authors focus on data from only one part of the dataset (building 0) and only choose the strongest RSSI signals. Literally, “In particular, the Building 0 from UJIIndoorLoc dataset is chosen to evaluate EdgeLoc and the top-40 APs (out of a total 520 APs) are selected.” Although AP selection can be performed as an optimisation step in this method, restricting evaluation to just one building makes the results not directly comparable with the baseline method or with other methods that used the full operational area.

The authors in [29] do not specify how the data were used. Literally, “In order to better verify the performance of the algorithm, we also conducted experiments on another widely used positioning dataset UJIIndoorLoc.” However, this algorithm requires a set of evaluation paths to asses the proposed algorithm, which is not provided in the original UJIIndoorLoc dataset. The full details about the data points used and how the evaluation paths were generated are lacking, i.e., the information provided does not enable reproducibility/replicability of the results.

In [83], the authors use the UJIIndoorLoc as an additional experiment alongside their main work. However, the dataset was restricted to a small subset of the UJIIndoorLoc dataset. Literally, “The database from two random phone users (phone id: 13 and 14) in two different buildings (building id: 0 and 1) are used.” In this way, the data to be analyzed and trained on are much simpler and similar, resulting in optimistic performance compared to the baseline method and other solutions using the whole UJIIndoorLoc dataset.

In [85], the authors used two datasets, including UJIIndoorLoc, to assess their proposed model. Despite providing details about the other dataset, they only mention that the UJIIndoorLoc dataset had 21,048 Wi-Fi fingerprints. It seems that the training and evaluation sets were merged into a common superset, which was later split by building ID in order to evaluate the model in three scenarios (buildings). Each of the three sets were split with a ratio of 70:30 to train and evaluate the proposed RDF ensembles.

In [86], the authors randomly split the training set into training and validation sets with a ratio of 80:20. Then, the resulting solution based on CNN was tested over the 1111 evaluation samples, as in the original dataset for the models based on single RSS readings. However, for the method they proposed based on multiple consecutive RSS readings, they had to manipulate the original dataset, splitting the original training set into training, validation, and testing sets with 60%, 20% and 20% of data from the original training set, respectively, i.e., the proposed method was not assessed over an independent test set.

Finally, the original division for training and evaluation provided in UJIIndoorLoc was followed in [81,84]. In [21], the authors do not detail how they trained their model with the UJIIndoorLoc dataset, as they only mention that the dataset contains 21,049 fingerprint samples. Although there is no clear indication about which data were used for training and evaluation, the context provided in the paper suggests that the authors used the evaluation data properly.

To sum up, the UJIIndoorLoc dataset includes a set for training and a set to test the accuracy of an IPS based on Wi-Fi fingerprinting. However, several authors mixed the two sets to apply cross-validation in order to create their own training, validation, and/or test sets, which led to data leakage. In these cases, overly optimistic results were obtained in validation and testing, as the subsets were not independent. In addition, we have observed that full details are often not provided when reporting experiments, which does not enable full reproducibility or replicability of research.

### 5.6. Experimental Scenarios

**Figure 9:**
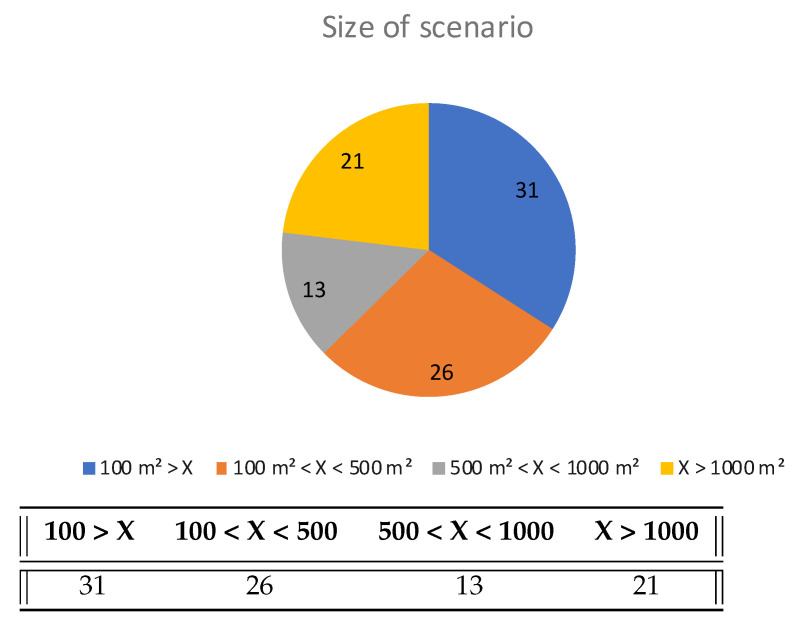
Size of scenarios used in experiments (in square meters).

Regarding the scenarios in which experiments take place, there is a great diversity of areas. The spaces range from universities to parking lots, stores, residential buildings, etc. As can be seen in Figure 9, 31 articles used an area of less than 100 $m^{2}$, 26 between 100 $m^{2}$ and 500 $m^{2}$, 13 between 500 $m^{2}$ and 1000 $m^{2}$, and 21 higher than 1000 $m^{2}$. It is important to highlight that in scenarios smaller than 100 $m^{2}$, the use of rooms drops to 41%. In the overall studies, this value is 66%. Although it is true that there are experiments in very small spaces, in general this value indicates that precision is usually prioritized over realistic environments. It should be noted that there are articles with more than one experiment and/or simulation, and every area in these papers is counted here.

Another important aspect of Wi-Fi fingerprinting radio maps is whether they are used in spaces without rooms or with rooms. This simple fact can greatly change the results of a study. Specifically, 103 of the radio maps used have rooms in their experimentation space, 53 were in spaces without any rooms (spaces without walls), and there are 5 papers that do not indicate any such parameter. Finally, it should be noted, again, that most of the works are performed with the focus on obtaining the best results, and not on performing experiments in a realistic environment. For example, changes in RSSI signals are not significant in experiments of short duration and in very delimited spaces.

## 6. Conclusions

In this paper, we have shown an analysis of the use of machine learning for indoor positioning in Wi-Fi based systems. The starting point has been a systematic review, following the PRISMA guidelines, of the current status of the application of deep learning algorithms applied to indoor positioning using Wi-Fi. Information from 119 articles published between 2016 and 2021 has been extracted and analyzed. In total, 161 simulations or experiments were analyzed.

In this study, we observed a tendency to use Neural Networks in solutions based on the use of Wi-Fi networks. However, we did not find any optimal or standard configuration. In addition to Neural Networks, SVM is widely used as well.

We noted the predominant use of RSSI Wi-Fi signals, although the studies that focus on the CSI are very promising, and are the ones that have obtained the best accuracy; furthermore, in the last year there has been an increase in the number of articles focusing on the use of this information. The only drawback is the difficulty of accessing this information on a Wi-Fi signal.

In analyzing the quality of the results, the Mean Error is the most widely used metric, followed by Accuracy (in percentage). In all cases, the articles analyzed in this review provided results followed the ISO/IEC 18305 guidelines.

Regarding their experiments, we found that most of the papers used empirical results. These papers usually show better results, however, this is generally due to better prepared environments. One important element that we found is that most of the papers prioritize improved results instead of working in a real environment. Thus, although test field sizes range from $6.7\;m^{2}$ to $10,000\;m^{2}$, many of these experiments are performed in small work spaces with many reference points and/or APs, or in open spaces without walls, which leads to unrealistic results in everyday environments. Likewise, we found studies in which training and validation data are misused by using repeated values for both sets.

On the other hand, forty papers used public datasets, among which the most popular was UJIIndoorLoc. Using public datasets allows for comparisons to be made between algorithms, as they are tested in the same environment. However, a deep analysis on how UJIIndoorLoc has been used revealed that many authors created their own test and validation data from the training dataset, which leads to overfitting and therefore, to better results than would be obtained with the baseline dataset.

The tables included in this review should be useful for those who want to focus their work based on the size of their work area, choice of machine learning algorithms, and desired accuracy, as well as their choice of the currently most commonly used metrics.

## Figures and Tables

**Table 1:** Public dataset summary.

Public Radio Map	Year	Size	APs	rPoints	Others
UJIIndoorLoc	2014	110,000 $m^{2}$	520	993	three buildings with four or five floors depending on the building.
IPIN2016	2016	150 $m^{2}$	168	57	a university corridor
UTSIndoorLoc	2019	44,000 $m^{2}$	589	1452	a building with sixteen floors, including three basement levels
JUIndoorLoc	2019	2646 $m^{2}$	172	2646	faculty rooms, classrooms, seminar rooms, research labs, and corridor
Rajen Bhatt	2019	4 rooms	7	1000	conference room, kitchen, or indoor sports room
Cramariuc	2016	2 university building	663	2651	data divided into two different University buildings.
WiFine	2020	9000 $m^{2}$	436	26,418	based on 260 trajectories
UJI Library	2020	308.4 $m^{2}$	448	212	data taken across fifteen months at the same positions and directions
Tampere	2017	22,570 $m^{2}$	992	4648	882 rooms on six floors

**Table 2:** Articles that used the UJIIndoorLoc dataset.

Art	Year	mAlg	mError
[41]	2021	DRL	3.06 m
[80]	2021	CHISEL (CNN)	6.95 m
[21]	2021	CNN	12.4 m
[81]	2021	DeepLocBox (NN)	9.07 m
[82]	2021	Edgeloc(CapsNet)	7.93 m
[29]	2020	RNN	4.91 m
[83]	2019	RNN	4.2 m
[84]	2019	MLP	5.64 m
[85]	2018	RDF	6.72 m
[86]	2018	CNN—Single RSS vector	10.25 m
		CNN—Time Series	2.77 m

## Abbreviations

The following abbreviations are used in this manuscript:

ANN	Artificial Neural Networks
AoA	Angle of Arrival
AP	Access Point
BPNN	Back Propagation Neural Network
CapsNet	Capsule Neural Network
CNN	Convolutional Neural Networks
CSI	Channel State Information
DBN	Deep Belief Network
DNN	Deep Neural Networks
DQN	Deep Q-Networks
DRL	Deep reinforcement learning
ELM	Extreme learning machine
GNSS	Global Navigation Satellite Systems
MLP	Multilayer Perceptron
MSE	Mean Squared Error
NN	Neural Network
PoA	Phase of Arrival
RF	Random Forest
RMSE	Root Mean Squared Error
RNN	Recurrent Neural Networks
RSSI	Received Signal Strength Indicator
SDA	Stacked Denoising Autoencoders
SMN	Single Multiplicative Neuron
SNR	Signal-to-Noise Ratio
SVM	Suport Vector Machines
ToF	Time Of Flight
VAE	Variational Autoencoder

## Appendix A. Full Features of Reviewed Articles

**Table A1:** Summary of reviewed articles.

Art	Year	Est	AP	rPoint	fMap	fmRoom	mAlg	sAlg	mError	oError	sType
[41]	2021	pMap	168	57	IPIN2016	N	DRL		0.92 m		RSSI
		pMap	589	1452	UTSIndoorLoc	Y	DRL		1.72 m		RSSI
		pMap	520	993	UJIIndoorLoc	Y	DRL		3.06 m	Only Building B1	RSSI
[22]	2021	pMap	96	80	JUIndoorLoc	Y	BayesNet	Dempster–Shafer		Accuracy = 80% between 3 and 3.6 m	RSSI
		pMap	520	993	UJIIndoorLoc	Y	BayesNet			Accuracy = 98% in 2 m	RSSI
[87]	2021	exp	7	116	1052 $m^{2}$	Y	SISAE (NN)		1.93 m	std = 1.34 m	RSSI
[44]	2021	exp	1	32	$49.9\;m^{2}$	N	CNN		1.76 m		CSI
		exp	1	45	40 $m^{2}$	N	CNN		1.16 m		CSI
		exp	1	66	$48.8m^{2}$	Y	CNN		2.54 m		CSI
		exp	1	15	32 $m^{2}$	N	CNN		0.91 m		CSI
[45]	2021	sim	15	158	1160 $m^{2}$	Y	ASDELM (ELM)			Accuracy = 85,90% in 1 m	CSI
		exp	22	47	384 $m^{2}$	Y	ASDELM (ELM)			Accuracy = 77% in 1 m	CSI
[88]	2021	pMap	520	993	UJIIndoorLoc	Y	DNNIP			Accuracy = 89% building and floor	RSSI
[80]	2021	pMap	520	993	UJIIndoorLoc	Y	CHISEL (CNN)	autoencoder	6.95 m	Accuracy = 99.6% building, 83.97% floor	RSSI
[46]	2021	exp	1	40	131.3 $m^{2}$	Y	BPNN	adaptive genetic algorithm		Accuracy = 90.47% in 4 m	CSI
[30]	2021	pMap	520	993	UJIIndoorLoc	Y	NNELILS (NN)			67% to 78% localization accuracies	RSSI
		pMap	96	80	JUIndoorLoc	Y	NNELILS (NN)		2.2 m to 2.6 m		RSSI
[89]	2021	pMap	309	3951	Tampere	Y	CMDRNN (cnn)		8.26 m	std = 1.31 m	RSSI
[21]	2021	pMap	520	993	UJIIndoorLoc	Y	CDAE i CNN		12.4 m		RSSI
		pMap	152	670	Alcala Tutorial 2017	N	CDAE-CNN		1.05 m		RSSI
[89]	2021	pMap	520	993	UJIIndoorLoc	Y	CMDRNN (cnn)		8.26 m	std = 1.31 m	RSSI
[90]	2021	exp	113	30	3600 $m^{2}$	Y	WiFiNet (cnn)			Accuracy = 91.89% in 2 m	RSSI
[81]	2021	pMap	520	993	UJIIndoorLoc	Y	DeepLocBox (NN)		9.07 m		RSSI
[33]	2021	exp	15	150	200 $m^{2}$	Y	SVM	M-LS	2.7 m		RSSI
[47]	2021	exp	1	N/A	14 $m^{2}$	N	NN		0.18 m		CSI
		exp	2	N/A	18 $m^{2}$	N	NN		0.03 m		CSI
		exp	2	N/A	6.7 $m^{2}$	Y	NN		0.08 m		CSI
[48]	2021	exp	1	317	148.5 $m^{2}$	Y	BLS(NN)		2.54 m		CSI
		exp	1	176	126 $m^{2}$	N	BLS(NN)		1.48 m		CSI
[82]	2021	exp	6	132	460 $m^{2}$	Y	Edgeloc(CapsNet)			99% under 2 m	RSSI
		pMap	520	993	UJIIndoorLoc	Y	Edgeloc(CapsNet)		7.93 m		RSSI
[91]	2021	exp	1	210	600 $m^{2}$	Y	MLR		4.03 m		RSSI
[77]	2021	exp	436	654	WIFINE	Y	RNN		3.05 m		RSSI
[92]	2021	exp	191	349	360 $m^{2}$	N	DNN		1.08 m		RSSI
[49]	2021	exp	1	17,486	CTW 2019 challenge	N	CNN		0.12 m		CSI
[93]	2021	exp	N/A	292	600 $m^{2}$	Y	CNN		1.86 m	Accuracy = 95% in 5.41 m	RSSI
		exp	N/A	262	1360 $m^{2}$	Y	CNN		1.86 m	Accuracy = 95% in 5.41 m	RSSI
[94]	2021	exp	12	680	6000 $m^{2}$	N	DNN		3.6 m		RSSI
		exp	12	170	6000 $m^{2}$	N	DNN		3.7 m		RSSI
		exp	12	40	6000 $m^{2}$	N	DNN		3.8 m		RSSI
[95]	2021	exp	4	54	69.35 $m^{2}$	Y	ANN			Accuracy = 13.84% < 0.5 m & 23.07% 0.5 < 1 m	RSSI
[50]	2020	exp	3	21	45 $m^{2}$	Y	CNN		1.27 m	std = 0.68 m	CSI
[36]	2020	exp	4	264	112 $m^{2}$	N	RF		1.68 m		RSSI
[51]	2020	exp	4	63	75.6 $m^{2}$	N	CNN		1.61 m		CSI
		exp	4	N/A	44.8 $m^{2}$	N	CNN		1.11 m		CSI
		exp	4	N/A	16 $m^{2}$	N	CNN		0.98 m		CSI
[96]	2020	exp	4	10	169 $m^{2}$	Y	CNN		0.98 m		RSSI
[54]	2020	exp	5	34	55 $m^{2}$	N	MLP	Regression	0.37 m	RMSE = 0.84 m	SNR

**art**: Article; **mAlg**: Main algorithm used; **est**: Experimental or pMapulated study; **sAlg**: Other algorithms used in the study; **AP**: APs used; **mError**: Mean Error; **rPoint**: Reference Points used in offline phase; **oError**: Other metrics reported in the study; **fMap**: Size of experimental room or radio-map used; **sType**: Signal type used; **fmRoom**: Rooms used in exp/pMap.

**Table A2:** Summary of reviewed articles.

Art	Year	Est	AP	rPoint	fMap	fmRoom	mAlg	sAlg	mError	oError	sType
[23]	2020	pMap	520	993	UJIIndoorLoc	N	KNN, LR, SVM, RF			RMSE = 1.87 m	RSSI
[55]	2020	exp	6	112	460 $m^{2}$	Y	capsnet		0.68 m		RSSI
[31]	2020	exp	8	133	512 $m^{2}$	N	Deep Fuzzy Forest		1.36 m	RMSE = 1.79 m	RSSI
[52]	2020	exp	1	32	50 $m^{2}$	N	CNN		1.77 m		CSI
		exp	1	24	40 $m^{2}$	N	CNN		1.16 m		CSI
		exp	1	66	49 $m^{2}$	N	CNN		2.54 m		CSI
[97]	2020	exp	6	50	60 $m^{2}$	N	RF	Bernoulli distribution		RMSE = 2.50 m	RSSI
[98]	2020	exp	25	240	315 $m^{2}$	N	RF	Co-forest	2.44 m		RSSI
		exp	5	N/A	NULL	N	RF		4.44 m		RSSI
[24]	2020	pMap	7	1000	Rajen Bhatt	Y	MLP			Accuracy = 94.4%	RSSI
[25]	2020	pMap	520	993	UJIIndoorLoc	Y	CNN			Accuracy = 88%	RSSI
[99]	2020	exp	195	300	800 $m^{2}$	N	DNN	HMM	1.22 m	RMSE = 1.43 m	RSSI
[32]	2020	exp	3	56	87.75 $m^{2}$	N	DNN	LC	0.78 m	std = 1.96 m	CSI
[28]	2020	exp	4	236	1148 $m^{2}$	Y	BPNN	GA-PSO	0.22 m		RSSI
[26]	2020	exp	10	102	568.4 $m^{2}$	Y	LSTM	LF-D	1.48 m		RSSI
		exp	30	353	2750 $m^{2}$	Y	LSTM		1.75 m		RSSI
[27]	2020	pMap	N/A	N/A	Cramariuc	Y	SEQ2SEQ	LSTM	5.5 m		RSSI
		pMap.	N/A	N/A	Cramariuc	Y	SEQ2SEQ		3.08 m		RSSI
[100]	2020	pMap	N/A	N/A	IPIN2016	Y	CNN, LSTM		4.93 m		RSSI
		pMap	N/A	N/A	IPIN2016	Y	CNN, LSTM		5.4 m		RSSI
		pMap	520	993	UJI Library	Y	CNN, LSTM		3.2 m		RSSI
		pMap	520	993	UJI Library	Y	CNN, LSTM		4.98 m		RSSI
[56]	2020	exp	5	22	293 $m^{2}$	Y	DNN			Accuracy = 95.45% in 3.65 × 3.65 m	RSSI
[29]	2020	exp	N/A	157	5500 $m^{2}$	Y	RNN	DL	3.05 m	std = 2.818 m	RSSI
		pMap	520	993	UJIIndoorLoc	Y	RNN		4.92 m	std = 3.719 m	RSSI
		sim	4	00	1681 $m^{2}$	Y	RNN	DL	2.42 m–2.92 m		RSSI
[101]	2020	sim	54	54	10,000 m ${}^{2}$	N	MLP		3.35 m		RSSI
[9]	2020	exp	3	7	25 $m^{2}$	Y	DNN	RESNET	0.11 m	RMSE = 0.08 m	SNR
[102]	2020	pMap	N/A	40	UJI Library	N	CNN	SVR	2.15 m		RSSI
[66]	2019	exp	3	30	540 $m^{2}$	N	DBN	cross entropy and the mean squared		NULL	RSSI
[34]	2019	exp	2	59	125 $m^{2}$	Y	SVM		0.7 m		RSSI
[57]	2019	exp	N/A	206	NULL	Y	DNN	Stacked AutoEncoder		Accuracy = 85%	RSSI
[35]	2019	exp	1	100	100 $m^{2}$	N	SVM		1.9 m	std = 0.07 m	CSI
[103]	2019	exp	N/A	N/A	NULL	NULL	CNN			RMSE = 0.31 m	RSSI
[104]	2019	exp	1	N/A	63 $m^{2}$	Y	SVM			96.4%	RSSI
		exp	1	N/A	63 $m^{2}$	Y	MLP			96.5%	RSSI
[53]	2019	exp	N/A	N/A	NULL	NULL	SVM			RMSE = 0.42 m	CSI
[58]	2019	exp	16	83	305 $m^{2}$	Y	DNN		2 m		RSSI
[59]	2019	pMap	520	993	UJIIndoorLoc	Y	CNN			Accuracy = 95.92%	RSSI
		pMap	309	3951	Tampere	Y	CNN			Accuracy = 94.13%	RSSI
[105]	2019	exp	6	300	300 $m^{2}$	N	MEA-BP		0.72 m		RSSI
[67]	2019	exp	50	N/A	NULL	NULL	ELM			NULL	RSSI
[61]	2019	exp	256	74	1664 $m^{2}$	Y	CNN			Accuracy = 95.4% in 4 m	RSSI
[62]	2019	exp	54	180	1209 $m^{2}$	Y	RDF			Accuracy = 89% at room level	RSSI
[64]	2019	exp	256	74	300 $m^{2}$	Y	CNN		1.46 m	Accuracy = 94% std = 2.24 m	RSSI

**art**: Article; **mAlg**: Main algorithm used; **est**: Experimental or pMapulated study; **sAlg**: Other algorithms used in the study; **AP**: APs used; **mError**: Mean Error; **rPoint**: Reference Points used in offline phase; **oError**: Other metrics reported in the study; **fMap**: Size of experimental room or radio-map used; **sType**: Signal type used; **fmRoom**: Rooms used in exp/pMap.

**Table A3:** Summary of reviewed articles.

Art	Year	Est	AP	rPoint	fMap	fmRoom	mAlg	sAlg	mError	oError	sType
[106]	2019	exp	4	42	80 $m^{2}$	N	RBF	LM	1.42 m	RMSE = 1.459 m	RSSI
[107]	2019	exp	N/A	300	302 $m^{2}$	Y	SVM		4.6 m		RSSI
[60]	2019	exp	5	10	NULL	N	RF			Accuracy = 97.5% in 2 m	RSSI
[108]	2019	exp	8	107	512 $m^{2}$	Y	K-ELM			RMSE = 1.7123 m std = 2.418 m	RSSI
[109]	2019	exp	9	96	560 $m^{2}$	Y	QKMMCC			average = 0.76m	RSSI
[65]	2019	pMap	520	993	UJIIndoorLoc	Y	RNN			Accuracy = 87.41%floor std = 0.83 m	RSSI
		exp	7	N/A	4 Rooms	Y	RNN			Accuracy = 95.8% std = 0.60 m	RSSI
[83]	2019	pMap	520	993	UJIIndoorLoc	Y	RNN		4.2 m	std = 3.2 m	RSSI
		exp	6	365	336 $m^{2}$	Y	RNN		0.75 m	std = 0.64 m	RSSI
[110]	2019	exp	9	261	300 $m^{2}$	N	BPNN		2.7 m	Accuracy = 90%	RSSI
[111]	2019	exp	8	66	736 $m^{2}$	Y	SDA		3.7 m	Accuracy = 84%	RSSI
[112]	2019	exp	1	42	50 $m^{2}$	N	CNN		0.46 m	without obstacles	RSSI
		exp	1	42	50 $m^{2}$	N	CNN		1.11 m	with some obstacles	RSSI
[113]	2019	exp	1	15	20 $m^{2}$	Y	MLP		1.42 m		RSSI
		exp	1	15	20 $m^{2}$	Y	CNN		1.67 m		RSSI
		exp	1	15	14.4 $m^{2}$	N	MLP		1.43 m		RSSI
		exp	1	15	14.4 $m^{2}$	N	CNN		1.51 m		RSSI
[114]	2019	exp	258	9	125 $m^{2}$	Y	CNN		3.91 m	Accuracy = 84%	RSSI
[63]	2019	pMap	N/A	N/A	NULL	Y	BPNN	ACO		Accuracy = 91.4%	RSSI
[115]	2019	pMap	520	993	UJI Library	Y	CNN, GRP		3.6 m	90% less 2m	RSSI
[42]	2019	exp	1	25	26.4 $m^{2}$	N	BPNN	PCA-PD	1.42 m	std = 1.1511 m	CSI
[84]	2019	exp	N/A	20	1200 $m^{2}$	Y	MLP	SDAE	3.05 m	1day	RSSI
		exp	N/A	57	2400 $m^{2}$	Y	MLP	SDAE	3.39 m	2 days	RSSI
		pMap	520	993	UJIIndoorLoc	Y	MLP	SDAE	5.64 m	10 days	RSSI
[116]	2019	pMap	520	993	UJIIndoorLoc	Y	VAE			RMSE = 4.65 m	RSSI
[117]	2019	exp	6	49	1600 $m^{2}$	Y	DNN		0.95 m	Open Doors	RSSI
		exp	6	49	1600 $m^{2}$	Y	DNN		1.26 m	Closed Doors	RSSI
[118]	2019	exp	4	228	1200 $m^{2}$	Y	ANN		1.22 m		RSSI
		exp	N/A	N/A	N/A	Y	ANN		1.90 m		RSSI
[119]	2019	exp	7	25	1728 $m^{2}$	N	RNN	LSTM	1.05 m	std = 0.8856 m	RSSI
[120]	2019	exp	15	71	4000 $m^{2}$	Y	NN	GA	3.47 m		RSSI
[121]	2019	exp	4	50	1100 $m^{2}$	Y	BGM		2.9 m		RSSI
[122]	2019	exp	122	48	629 $m^{2}$	Y	DNN		2.64 m		RSSI
		exp	59	139	65 $m^{2}$	N	DNN		1.21 m		RSSI
[123]	2018	pMap	520	993	UJIIndoorLoc	Y	CNN			95.76% floor level	RSSI
[124]	2018	pMap	7	1000	Rajen Bhatt	Y	RF			98.3% floor level	RSSI
[125]	2018	exp	20	2100	8250 $m^{2}$	Y	DNN		3.95 m	std = 2.72 m	RSSI
[126]	2018	exp	16	202	806 $m^{2}$	Y	SMN	PCA	1.85 m	std = 1.04 m	RSSI
[127]	2018	pMap	520	993	UJIIndoorLoc	Y	DQN			78.79% in 1 m	RSSI
[37]	2018	exp	50	180	75 $m^{2}$	Y	RF		1.29 m	90% in 3 m	RSSI
[128]	2018	exp	N/A	N/A	NULL	Y	DNN			83.6% floor with people, 99.6% without	RSSI

**art**: Article; **mAlg**: Main algorithm used; **est**: Experimental or pMapulated study; sAlg: Other algorithms used in the study; **AP**: APs used; **mError**: Mean Error; **rPoint**: Reference Points used in offline phase; **oError**: Other metrics reported in the study; **fMap**: Size of experimental room or radio-map used; **sType**: Signal type used; **fmRoom**: Rooms used in exp/pMap.

**Table A4:** Summary of reviewed articles.

Art	Year	Est	AP	rPoint	fMap	fmRoom	mAlg	sAlg	mError	oError	sType
[129]	2018	pMap	N/A	N/A	UJI Library	Y	RNN		2.48 m	99.6% floor level	RSSI
		pMap	N/A	N/A	UJI Library	Y	LSTM		2.6 m	99.5% floor level	RSSI
[85]	2018	pMap	520	993	UJIIndoorLoc	Y	RDF		6.72 m	std = 4.82 m	RSSI
[130]	2018	exp	7	101	404.5 $m^{2}$	Y	FF-DNN			RMSE = 0.32 m, 53.123% in 0.5 m	RSSI
[43]	2018	exp	4	25	80 $m^{2}$	N	RF		0.40 m		CSI
[131]	2018	exp	4	67	1664 $m^{2}$	Y	SVM		1.34 m		RSSI
[86]	2018	pMap	520	993	UJIIndoorLoc	Y	CNN		2.77 m	100% for floor prediction	RSSI
[132]	2018	exp	N/A	N/A	NULL	NULL	SVR	RBF Kernel		95% in 1.81 m	RSSI
[133]	2018	exp	40	180	1209 $m^{2}$	Y	RF			95% accuracy 1.5 × 1.5 m	RSSI
[134]	2018	exp	8	40	580 $m^{2}$	Y	RVFL		0.43 m	RMSE = 0.5830 m	RSSI
[69]	2018	sim	4	36	441 $m^{2}$	N	RVM	PLS	0.84 m		RSSI
		exp	6	25	156 $m^{2}$	Y	RVM	PLS		41% in 1 m and 91% in 2 m	RSSI
[135]	2017	exp	3	110	109.25 $m^{2}$	N	FF-DNN			RMSE = 0.6782 m	RSSI
[136]	2017	exp	4	N/A	NULL	N	ANN			RMSE = 1.1045 m	RSSI
		exp	6	N/A	NULL	N	ANN			RMSE = 1.2288 m	RSSI
[137]	2017	exp	16	126	304 $m^{2}$	Y	SVM		1.43 m		RSSI
[138]	2017	sim	6	441	100 $m^{2}$	N	LS-SVM		2.56 m		RSSI
[139]	2017	exp	38	411	600 $m^{2}$	Y	ELM		1.91 m		RSSI
[140]	2017	exp	28	67	30 $m^{2}$	N	ANN		2.2 m		RSSI
[141]	2017	exp	185	480	NULL	Y	SVM			100% shop level	RSSI
[142]	2017	pMap	520	993	UJIIndoorLoc	Y	DNN			92% floor recognition	RSSI
[143]	2017	exp	8	48	53.35 $m^{2}$	N	SVR			86.2% in 1.5 m and 90.4% in 2 m	RSSI
[144]	2017	exp	N/A	N/A	NULL	Y	SVM			97.31% flat and 88.38% floor	RSSI
[145]	2016	exp	22	84	387.75 $m^{2}$	Y	BPNN		0.98 m		RSSI
[146]	2016	sim	4	25	400 $m^{2}$	N	MLP-ANN		0.27 m	std = 0.36 m	RSSI
[147]	2016	sim	N/A	N/A	NULL	NULL	EB-ANN			RMSE = 0.4991 m	RSSI
[148]	2016	exp	5	54	150 $m^{2}$	Y	SVR			70% in 5 m	RSSI
[149]	2016	exp	16	188	1125 $m^{2}$	Y	ANN		1.89 m	90% in 2.971 m	RSSI
[70]	2016	sim	12	1600	1600 $m^{2}$	N	SVR			RMSE = 1.42 m	RSSI
		exp	13	116	1000 $m^{2}$	Y	SVR			RMSE = 1.8 m, 74% in 2 m	RSSI
[150]	2016	exp	N/A	112	460 $m^{2}$	Y	SVM		1.2 m		RSSI

**art**: Article; **mAlg**: Main algorithm used; **est**: Experimental or pMapulated study; sAlg: Other algorithms used in the study; **AP**: APs used; **mError**: Mean Error; **rPoint**: Reference Points used in offline phase; **oError**: Other metrics reported in the study; **fMap**: Size of experimental room or radio-map used; **sType**: Signal type used; **fmRoom**: Rooms used in exp/pMap.
